# Nomogram to Predict Occult N2 Lymph Nodes Metastases in Patients With Squamous Nonsmall Cell Lung Cancer

**DOI:** 10.1097/MD.0000000000002054

**Published:** 2015-11-20

**Authors:** Long Jiang, Shanshan Jiang, Yongbin Lin, Han Yang, Zehua Xie, Yaobin Lin, Hao Long

**Affiliations:** From the Sun Yat-sen University Cancer Center; State Key Laboratory of Oncology in South China; Collaborative Innovation Center for Cancer Medicine, Guangzhou, China (LJ, SJ, YL, HY, ZX, YL, HL); Lung Cancer Institute of Sun Yat-sen University, Guangzhou, China (LJ, YL, HY, ZX, YL, HL); Department of Thoracic Oncology, Sun Yat-sen University Cancer Center, Guangzhou, China (LJ, YL, HY, ZX, YL, HL); and University of California, San Francisco, San Francisco, CA (LJ).

## Abstract

For nonsmall cell lung cancer (NSCLC) patients without distant metastases, occult involvement of N2 lymph nodes would be of the utmost importance in determining both treatment and survival. The key to optimal treatment strategies relied on accurate diagnosis, in particular accurate clinical tumor staging. Patients with clinical N0 or N1 staging preoperatively had a sizeable risk to have occult N2 lymph nodes metastases.

From November 2004 to March 2007, the entire database in a tertiary hospital of all patients with a pathologic diagnosis of squamous NSCLC underwent anatomical pulmonary resection and systematic mediastinal lymph node dissection were retrospectively collected and reviewed. A nomogram was developed on the basis of a multivariable logistic regression model with a combination of all potential variables. In order to surmount the potential of overestimating predictive performance, both bootstrapping for internal validation and an independent external validation set were employed.

A nomogram incorporating the significant risk factors was created to predict the probability of occult N2 lymph nodes metastases. The calibration plot for the probability of occult N2 lymph nodes metastases showed an optimal agreement between the predicted probabilities by nomogram and actual observed probabilities. An objective and accurate nomogram predictive model for occult N2 lymph nodes metastases was drawn up and validated internally and externally in patients with squamous NSCLC.

The nomogram model, as a robust tool in predicting occult N2 lymph nodes involvement, could be involved in a cost-effective application of specific diagnostic and therapeutic strategies.

## INTRODUCTION

Lung cancer, the most commonly diagnosed cancer, has been the most frequent cause of cancer-related death in women and men throughout the world.^1^ Totally, nonsmall cell lung cancer (NSCLC) accounted for approximately 80% of lung cancers.^2^ Squamous NSCLC, revealing to be divergent clinical and molecular phenotypes compared with nonsquamous NSCLC, is hoped to be explored and confirmed to develop relative approach in managing squamous NSCLC.^3^ For patients without distant metastases, occult involvement of N2 lymph nodes would be of the utmost importance in determining both treatment and survival.^4^ Due to high risk of distant or local relapse in locally advanced NSCLC patients with N2 lymph node involvement, the optimal treatments for these patients were examined recently.^5^ The above reasons made precise and accurate preoperative mediastinal staging particularly important in potentially surgical candidates.^6^

In real world clinical routines, in many centers, patients with negative computed tomography (CT) or F-18-fluoro-2-deoxy-d-glucose positron emission tomography combined with thoracic computed tomography (FDG-PET/CT) mediastinal image would proceed directly to the thoracotomy.^7^ However, patients with NSCLC presenting with clinical N0 or N1 on CT or FDG-PET/CT staging preoperatively had a sizeable risk to have documented malignant mediastinal nodal diseases (occult N2 lymph nodes metastases).^8^ Remarkably, survival in patients with occult N2 lymph nodes metastases was poorer than those without, but similar to clinically suspected N2 disease.^9^ Thus, the attempt in determining risk factors associated with occult N2 lymph nodes metastases in patients with NSCLC is emerging, aiming in selecting candidates for sparingly and appropriately preoperative either cervical mediastinoscopy or endobronchial ultrasound transbronchial needle aspiration (EBUS-TBNA) based on accurate prediction, as well as determining cost-effective strategies of applying invasive or expensive diagnostic procedures.^10^

This retrospective study was undertaken to identify the actual risk factors of occult N2 lymph nodes metastases among patients with squamous NSCLC. A nomogram prediction model, which was validated both internally and externally, was intended to estimate the pretest probability of occult N2 lymph nodes metastases in patients with squamous NSCLC. An essential value of this model lies in its ability to estimate the pretest probability, which could then be used to refine the subsequent indication of lymph node staging modalities. Accurate diagnosis could avoid futile resection, unnecessary invasive staging, and potentially recruiting advanced patients as early stage, such as recently controversy focusing on stereotactic ablative radiotherapy trials.^11^ To our knowledge, this study is the first attempt to establish a predictive nomogram for occult N2 lymph nodes metastases among patients with squamous NSCLC based on the clinicoradiological data. In addition, a separate cohort was used for external validation.

## METHODS

Study protocol was approved by the institutional review boards of Sun Yat-Sen University Cancer Center (SYSUCC). Written informed consent was issued by each patient: including signed consent for tissue analysis as well as consent to be recorded for potential medical research at the time of sample acquisition. All experiments were carried out in accordance with relevant guidelines and regulations.

From November 2004 to March 2007, the entire database in a tertiary hospital of all patients with a pathologic diagnosis of squamous NSCLC underwent anatomical pulmonary resection and systematic mediastinal lymph node dissection at SYSUCC were retrospectively collected and reviewed. As clinical routine, contrast-enhanced chest and upper abdominal CT scans or FDG-PET/CT scan were performed within 1 month in SYSUCC before surgery for all patients, including those who had received CT scans in other hospitals. Other routine preoperative assessments included laboratory test parameters, bronchoscopy, chest radiography, brain magnetic resonance imaging, bone scan, cardiopulmonary function tests, and a cardiovascular-risk assessment. If needed, lung perfusion scans were also performed. Relative medical records were retrospectively reviewed for demographic and clinical data. All patients with lung cancer being taken into account in treatment at SYSUCC were discussed during a multidisciplinary tumor board meeting. Clinical stages were determined after chest CT and FDG-PET/CT followed the revised tumor-node-metastasis classification system of the American Joint Committee for Cancer Staging and Revised International System for Staging Lung Cancer.^9^ All collected information, including clinical, operative, radiological, and pathological records, were retrospectively reviewed and confirmed through reviewing departmental reports and radiological imaging. Pathological diagnosis was based on the World Health Organization classification.^12^ Clinicoradiological data on age, sex, height, weight, history of cancers, family history of cancers, history of other lung diseases (including chronic obstructive pulmonary disease and pulmonary fibrosis),^13^ smoking history, clinical stage, clinical T stage, clinical N stage, tumor size, tumor location, preoperative serum carcinoembryonic antigen (CEA) and squamous cell carcinoma antigen (SCC) level, and pathological diagnosis were collected for both patients in training or validation group.

Inclusion criteria were local squamous NSCLC with clinical N0 or N1 stage, in other words, without suspicious N2 or N3 disease on FDG-PET/CT or CT, or synchronous distant metastasis. Accordingly, exclusion criteria were patients with adenocarcinoma according to the new lung adenocarcinoma classification^14^; patients received neoadjuvant chemotherapy, target therapy, or radiotherapy; patients undergone surgical treatment previously; patients undergone mediastinoscopy alone or lymph node sampling during operation; patients without either FDG-PET/CT or chest CT; and patients without acquirable complete data.

The propensity score matching (PSM), aiming to overcome the differential distribution of potential risk covariates in different groups, was generated using all reported covariates with one-to-one nearest neighbor matching algorithm with a caliper of 0.2. The included characteristics as covariates were sex, age, height, weight, history of cancers, family history of cancers, history of other lung diseases, smoking history (measured by pack-year), clinical stage, clinical T stage, clinical N stage, tumor size, tumor location, central location, CEA, and SCC. The standardized difference in means and distribution of propensity scores was used in assessing the improvement of covariance balance after PSM. In total, 146 patients were enrolled into a training group, whose clinical data were analyzed to create the mathematical model. Clinical data were also collected from an additional matched 73 patients who were enrolled with the same inclusion criteria between April 2007 and March 2008 as validation group.

A lymph node would be considered as positive if its short axis exceeded 1 cm on chest CT images or it was positive on FDG-PET/CT images. All positive lymph nodes were localized based on the new International Association for the Study of Lung Cancer Lymph Node Map.^15^

Occult N2 lymph node metastasis was defined as an intraoperative or postoperative pathologically positive mediastinal lymph node in clinical N0–1 patients by FDG-PET/CT or chest CT.

A centrally located tumor was defined as the center of the tumor locating in the medial third of the lung parenchyma, while peripherally as the lateral two-thirds.^16^

## STATISTICAL ANALYSES

Categorical variables were presented as frequencies and percentages, while continuous variables as medians and ranges, unless otherwise stated. All available demographics and preoperative characteristics were tested for balance between training and validation group. The Pearson Chi-squared test or Fisher exact test was used in categoric variables. Accordingly, independent sample *t* test, analysis of variance, or Mann–Whitney *U* tests was used to evaluate continuous variables and discrete numerical variables.

### Construction of the Nomogram

In the training set, a nomogram was developed by using the package of rms on the basis of a multivariable logistic regression model with a combination of all potential variables, including sex, age, height, weight, history of cancers, family history of cancers, history of other lung diseases, smoking history (measured by pack-year), clinical stage, clinical T stage, clinical N stage, tumor size, tumor location, central location, CEA, and SCC. Variables would enter into the binary logistic regression analysis to form the prediction model if with a *P* value < 0.2 in univariate analyses.^17^ Forward stepwise selection procedures were used with the likelihood-ratio test to overcome the limitations of the stepwise selection method. Final selection for factors remaining in nomogram model was based on a backward step-down process with the Akaike information criterion as a stopping rule.^18^

### Validation and Calibration of the Nomogram

In order to overcome the potential of overestimating predictive performance, both bootstrapping for internal validation and an independent external validation set were employed.^19^ One thousand bootstrap samples were drawn, each sample size of which equaled to the original (146 in the present study), by randomly sampling 146 subjects with replacement from the original samples. Additionally, the nomogram model was validated using a separate data set of 73 patients after PSM. When externally validating the nomogram, the total points were computed according to the established nomogram for each patient in the validation cohort. Calibration of the nomogram model, defined as concordance between predicted and observed probabilities, was established with the Hosmer–Lemeshow goodness-of-fit test (*P* > 0.05) and the calibration plot exhibiting observed versus predicted probabilities. The discriminative ability of the predictive nomogram model was assessed by calculating the concordance index (C-index), whose value ranged from 0.5 (no discrimination) to 1.0 (perfect discrimination).^20^

Data management and statistical analyses were done with commercially available programs, IBM SPSS Statistics (IBM SPSS Statistics for Windows, Version 22.0. IBM Corp., Armonk, NY) for Windows (SPSS, Inc., Chicago, IL) and R (version 3.2.0; R Foundation for Statistical Computing, Vienna, Austria). All tests were 2-tailed and considered statistically significant if *P* < 0.05.

## RESULTS

**FIGURE 1:**
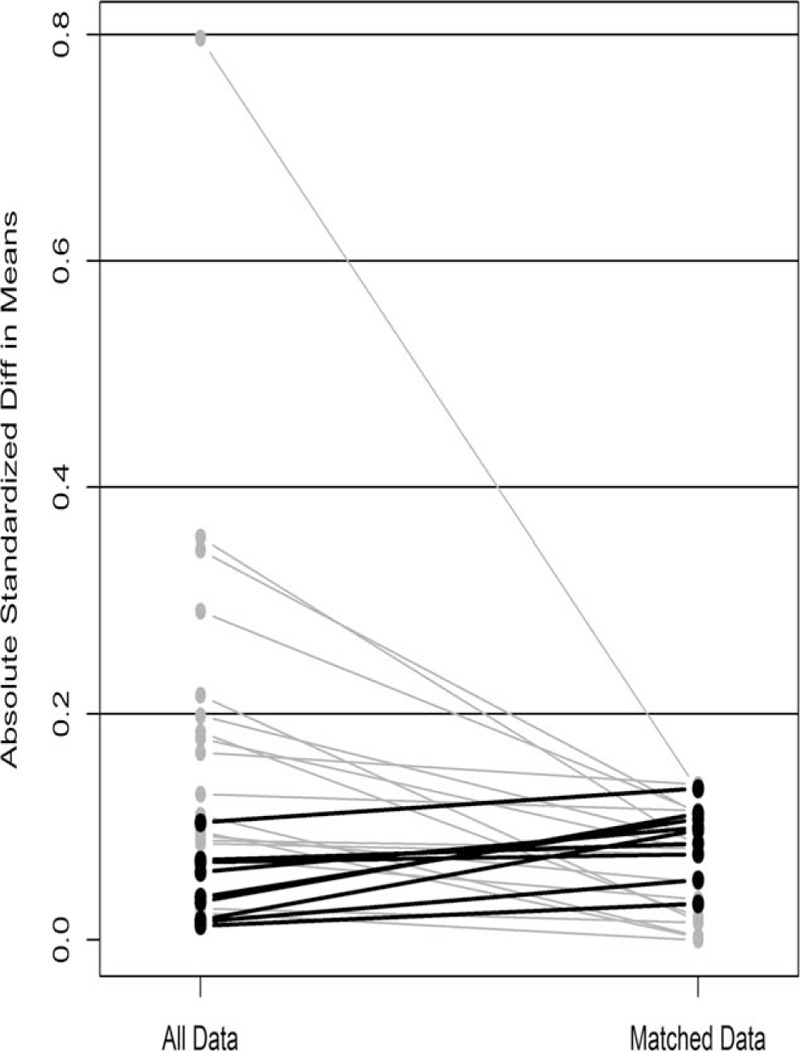
Parallel line plot of the standardized difference in means before and after propensity score matching in patients with squamous nonsmall cell lung cancer. As the standardized difference in means was reduced, covariate balance was improved in the matched samples.

**FIGURE 2:**
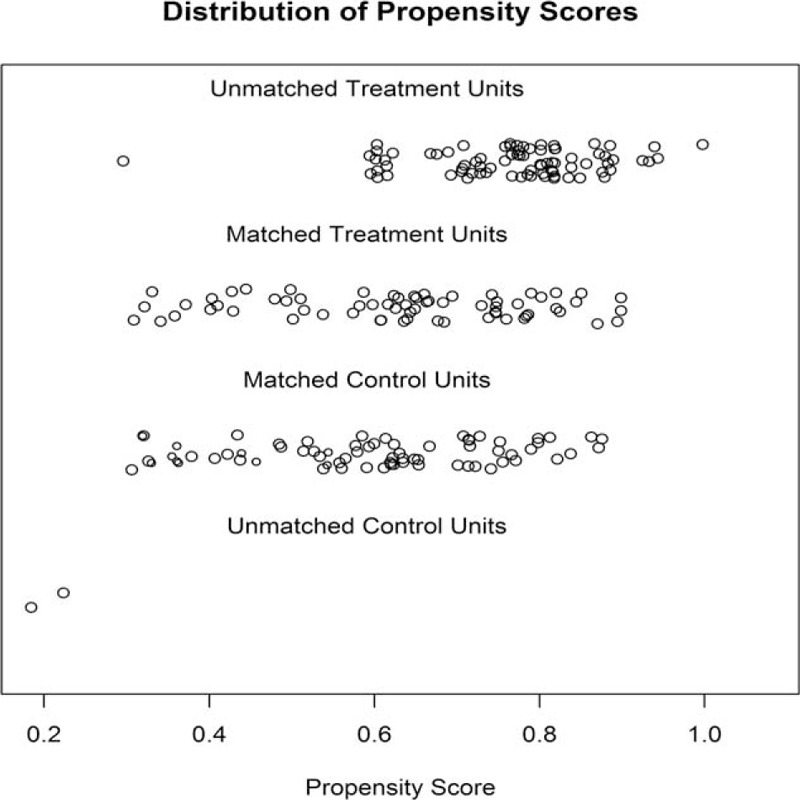
Dot plot of the propensity scores of patients with squamous nonsmall cell lung cancer showing individual units in the dataset and whether they were matched or discarded.

A total of 219 patients (146 in the training cohort and 73 in the validation cohort) fulfill the inclusion criteria and were enrolled to develop and validate our predictive nomogram model. Patient preoperative clinicoradiological characteristics are shown in Table 1. Two hundred patients were male (91.3%), while 19 patients were female (8.7%). The median age at diagnosis was 61 years (range, 32–80 years). Tumor size ranged from 0.5 to 12.0 cm (median, 4.0 cm). Smoking history, measured by pack-year, ranged from 0 to 180 (median 30). Moreover, the median height was 163 cm (range, 140–178 cm), while median weight 58 kg (range, 40–85 kg). Additionally, history of cancers presented in 8 patients (3.7 %), and family history of cancers in 31 patients (14.2 %), then history of other lung diseases in 25 patients (11.4%), along with central location in 69 patients (31.5%). Furthermore, the most common lobar location was the left upper lobe (27.9%), and most common clinical stage IB (32.9%), then the most common clinical T stage T2a (40.2%), along with the most common clinical N stage N0 (81.3%). Serum CEA, and SCC, ranged from 0.47 to 215.30 ng/mL (median 3.25 ng/mL) and 0.0 to 18.5 mg/mL (median 0.3 mg/mL), respectively. In addition, CT was performed in all patients (100%), while FDG-PET/CT scan in 101 patients (46.1%). Finally, a total of 219 surgical resections were performed for primary lung cancer. These constituted 174 lobectomies and 45 pneumonectomies resections. Surgical techniques were surgeons dependent, but unit policy was to undertake systematic lymph nodes dissections. Eventually, 3720 lymph nodes were dissected in all 219 patients, including station 2L (n = 38), station 2R (n = 301), station 3 (n = 78), station 4L (n = 69), station 4R (n = 315), station 5 (n = 270), station 6 (n = 79), station 7 (n = 789), station 8 (n = 50), station 9 (n = 226), station 10 (n = 423), and remaining N1 lymph nodes removed as part of the resection specimen (n = 1082). The mean number of lymph nodes dissected in each patient was 17.0. The incidence of occult N2 lymph nodes metastases in the present data set was 16.9% (37/219). All the above clinical characteristics were well balanced between training and validation cohort (Table 1). The standardized difference in means before and after PSM illustrated improvement of covariance balance through PSM procedure (Fig. 1). Consistently, this improvement was also proved by the distribution of propensity scores before and after PSM (Fig. 2).

**TABLE 1:**
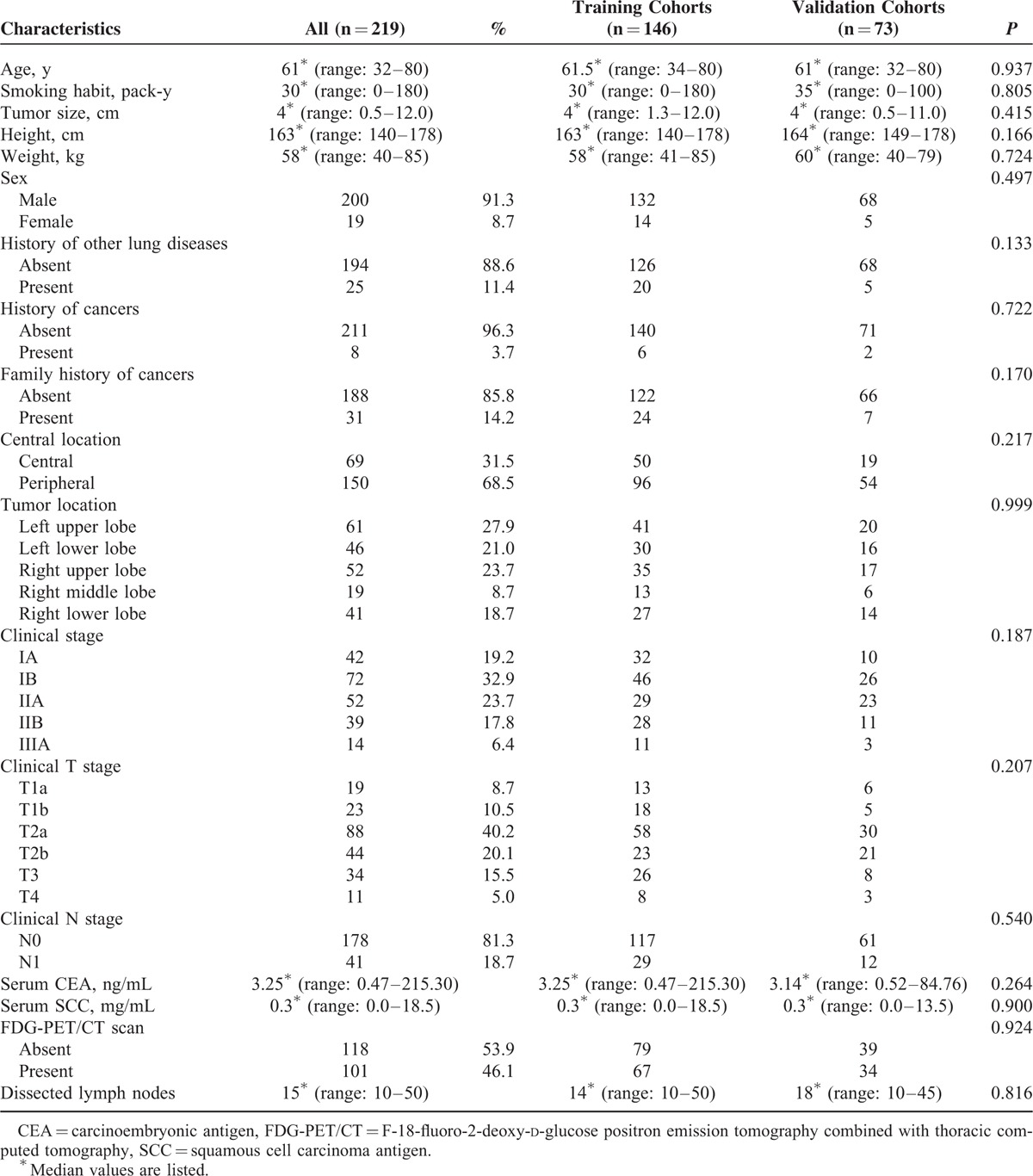
Clinicoradiological Characters in Training and Validation Cohorts

In univariate analysis of the training cohort (Table 2), occult N2 lymph nodes metastases were significantly correlated with sex (*P* = 0.001), age (*P* < 0.001), height (*P* = 0.183), weight (*P* = 0.484), history of cancers (*P* = 0.031), family history of cancers (*P* = 0.009), history of other lung diseases (*P* = 0.003), smoking history (*P* = 0.023), clinical stage (*P* = 0.047), clinical T stage (*P* = 0.003), clinical N stage (*P* = 0.009), tumor size (*P* = 0.020), tumor location (*P* = 0.006), central location (*P* = 0.021), CEA (*P* = 0.006), and SCC (*P* = 0.593).

**TABLE 2:**
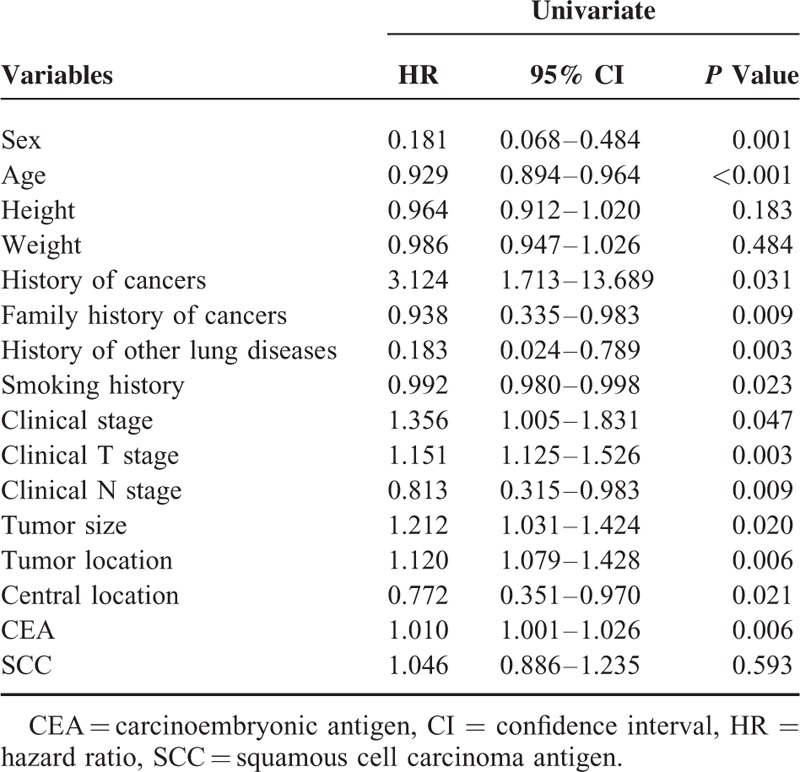
Univariate Analysis of Risk Factors for Occult N2 Lymph Nodes Metastases in Patients With Squamous Nonsmall Cell Lung Cancer

In multivariable analysis of the training cohort (Table 3), occult N2 lymph nodes metastases were significantly correlated with sex (*P* = 0.031), age (*P* = 0.001), history of cancers (*P* = 0.041), family history of cancers (*P* = 0.047), history of other lung diseases (*P* = 0.037), smoking history (*P* = 0.042), clinical stage (*P* = 0.045), clinical T stage (*P* = 0.033), clinical N stage (*P* = 0.029), tumor size (*P* = 0.037), tumor location (*P* = 0.026), central location (*P* = 0.034), and CEA (*P* = 0.007).

**TABLE 3:**
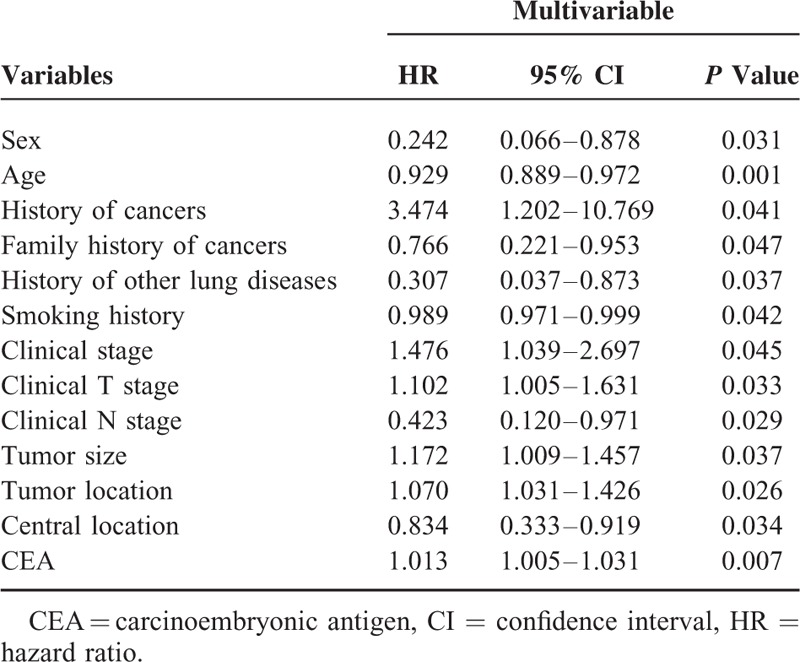
Multivariable Analysis of Risk Factors for Occult N2 Lymph Nodes Metastases in Patients With Squamous Nonsmall Cell Lung Cancer

### Predictive Nomogram for the Probability of Occult N2 Lymph Nodes Metastases

**FIGURE 3:**
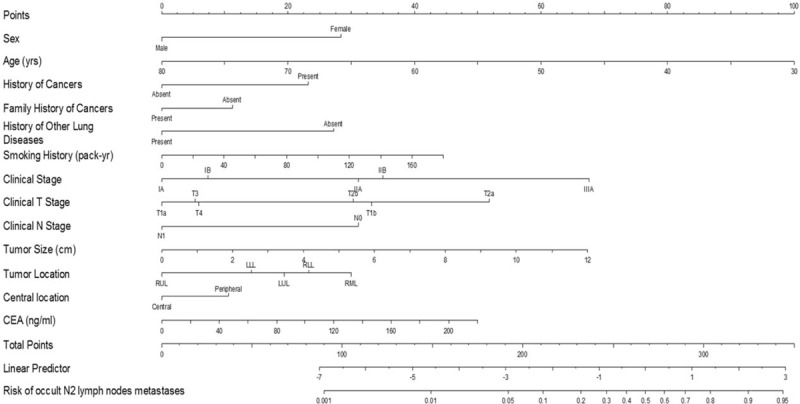
Predictive nomogram of occult N2 lymph nodes metastases for patients with squamous nonsmall cell lung cancer. LLL = left lower lobe, LUL = left upper lobe, RLL = right lower lobe, RML = right middle lobe, RUL = right upper lobe.

On the basis of the multivariable logistic regression of the training cohort, a nomogram incorporating the significant risk factors was set up to predict the probability of occult N2 lymph nodes metastases (Fig. 3). A total score was calculated using sex, age, history of cancers, family history of cancers, history of other lung diseases, smoking history, clinical stage, clinical T stage, clinical N stage, tumor size, tumor location, central location, and CEA. Each value of these variables was allocated a score on the point scale axis. For example, tumor location at right upper lobe (RUL) was 0 point and right lower lobe was 30 points. Interestingly, the allocated scores of clinical T stage showed that patients with T2a might be more likely to be occult N2 than patients with T3 or T4. A possible reason would be that patients with T3 or T4 might have already been diagnosed as clinical N2 diseases, instead of clinical N0 or N1.

A total score could be easily calculated by summing each single score and located on the total point scale axis. This total score could then be employed to assign the probability of occult N2 lymph nodes metastases for individual patients by drawing a straight line down to the scale axis at the bottom of the figure. Hosmer–Lemeshow test of goodness-of-fit revealed a high concordance between the predicted and observed probabilities (*P* = 0.784). In the meantime, this goodness-of-fit test indicated that excluding any 1 of these parameters would result in significant degradation of the predictive power of the nomogram model.

### Internal and External Validation for Predictive Accuracy of the Nomogram

**FIGURE 4:**
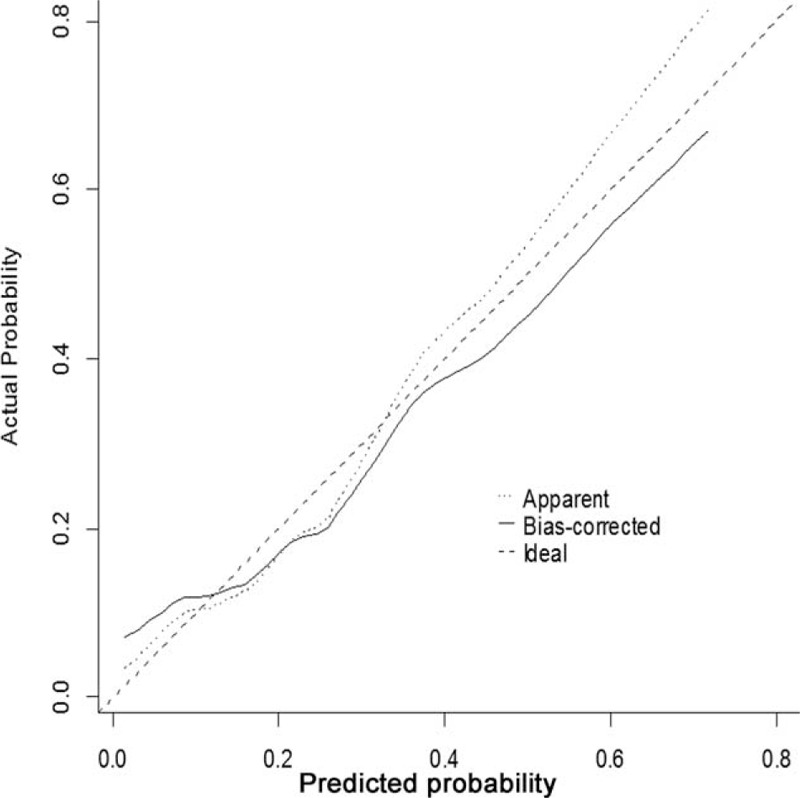
Calibration of the present nomogram model for predicting occult N2 lymph nodes metastases based on clinicoradiological variables. The x-axis represents the nomogram-predicted probability and the y-axis represents the observed rate of occult N2 lymph nodes metastases. Perfect prediction would correspond to the 45° dashed line. The dotted line represents the entire validation cohort (n = 73) and the solid line is bias-corrected by bootstrapping, indicating the observed nomogram performance.

**FIGURE 5:**
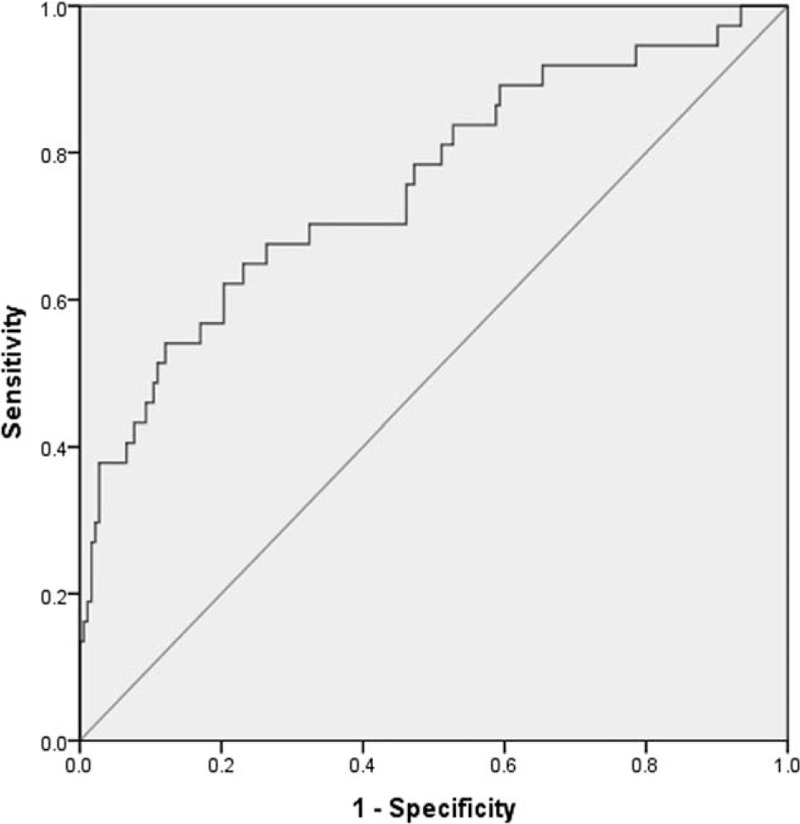
Receiver-operating characteristic curves of the models to predict occult N2 lymph nodes metastases [area under the ROC curve (AUC) = 0.756].

The nomogram was applied into both training and validation cohort for internal and external validation. The calibration plot for the probability of occult N2 lymph nodes metastases showed an optimal agreement between the predicted probabilities by nomogram and actual observed probabilities, indicating superior predictive power of the nomogram when applied to an independent validation data set (Fig. 4). The area under the receiver-operating characteristic (ROC) curve (Harrell's C-index for prediction), corresponding to the model's accuracy, was 0.756 (95% confidence interval, 0.662–0.850; Figure 5). Internal validation by bootstrapping showed the bias-corrected C-index for prediction was 0.830.

## DISCUSSION

This retrospective study, after identifying the risk factors of occult N2 lymph nodes metastases among patients with squamous NSCLC, established a nomogram prediction model to calculate the pretest probability of occult N2 lymph nodes metastases, with both internal and external validations.

In order to formulate optimal staging strategies, it is necessary to obtain the pretest probability of lymph node metastases, especially after the success of neo-adjuvant chemotherapy in patients with N2 diseases.^21^ Base on this purpose, the present study was undertaken with the attempt in calculating the probability of N2 lymph node metastases for individuals. In addition, as the consequence of the present study revealed, similar with previous study, 16.9% of patients were proved to be occult N2 disease after resection.^22^ This is why calculating the probability of N2 lymph node metastases for individuals is the key to justifying the proper preoperative management.

Similarly with previous reports, sex,^23^ age,^24^ tumor size,^25^ central tumor location,^22^ tumor location,^22^ and clinical N stage ^26^ were included and identified as risk factors for occult N2 lymph nodes involvement in the present study.

However, compared with previous investigations, the present nomogram model took the advantages as generating a predictive model to calculate the pretest probabilities for individuals, including more potential risk factors that were easily obtainable in clinical routines, high percentage of CT (100%) or FDG-PET/CT (46.1%) evaluating in clinical staging, applicable for all surgical candidates, reducing the limitations of selection bias from retrospective cohort by employing PSM method in recruiting procedure, validating by an independent dataset to avoid overfitting of the model and determine generalizability,^27^ optimal agreement between prediction and actual observation showed by C-index and calibration plots that guaranteed the reliability and repeatability of the established model, recruiting a relative large number of squamous NSCLC patients, who were never studied for occult N2 lymph nodes metastases independently. Nevertheless, due to the reason that only a small portion of squamous NSCLC patients were females,^28^ there were only few females subjects in both training and validation cohort in the present study. Therefore, further study with more female patients would be necessary to validate this nomogram in identifying occult N2 disease.

The present nomogram model could be applied directly in patients with clinical N0 or N1 NSCLC as diagnostic tools in predicting the pretest probability of N2 lymph nodes metastases. The quantified probability allowed clinicians to make more objective decisions and provided more convenient options in decision-making processes. For example, a hypothetical 70-year-old female and nonsmoker patient who has a peripherally RUL located squamous NSCLC, 2 cm in diameter and no suspected positive lymph nodes thus clinically staged as cT1aN0 IA, also absent of history of cancers but present with family history of cancer and history of other lung disease, and serum CEA of 80 ng/mL, we calculate a score of −5.75, and therefore the predicted probability of N2 lymph nodes metastases is 0.5%, which is low enough for the patient to go directly to surgery without further invasive diagnostic tests. Nevertheless, for patients with moderate risk of N2 lymph nodes metastases (eg, 15%), further invasive diagnostic tests, such as mediastinoscopy, should be recommended because the yields of mediastinoscopy would be <100,000 dollars per life-year gained when the risk of N2 lymph nodes involvements exceeding 10%.^29^ However, thresholds for N2 predicted probability was not definite and might vary among clinicians. Thus, the current nomogram model only provided an objective reference value aiming to aid decision-making process of management strategies. Moreover, although satisfactory predictive power of the present nomogram was validated internally and externally, it is still necessary to follow guideline in routine clinical practice, including mediastinoscopy, mediastinotomy, EBUS, Endoscopic ultrasound, and CT-guided biopsy for pathologic mediastinal lymph node evaluation,^13^ before validation from a prospective clinical trial. Whereas, it would be justified to apply the current nomogram into proper clinical trial.

In conclusion, an objective and accurate nomogram predictive model for occult N2 lymph nodes metastases was drawn up and validated internally and externally in patients with squamous NSCLC. The nomogram model, as a robust tool in predicting occult N2 lymph nodes involvement, could be implicated in a cost-effective application of specific diagnostic and therapeutic strategies. Additionally, this model enabled both clinicians and patients perform an individualized prediction through this easy-to-use predictive system. Furthermore, this nomogram also provided precious information in clinical trial design to gather better equivalence between study arms.
